# The High Q Factor Lateral Field–Excited Thickness Shear Mode Film Bulk Acoustic Resonator Working in Liquid

**DOI:** 10.3390/mi7120231

**Published:** 2016-12-14

**Authors:** Da Chen, Wenwen Ren, Shuren Song, Jingjing Wang, Weihui Liu, Peng Wang

**Affiliations:** 1 State Key Laboratory of Mining Disaster Prevention and Control Co-founded by Shandong Province and the Ministry of Science and Technology, Shandong University of Science and Technology, Qingdao 266590, China; 2 College of Electronic, Communication and Physics, Shandong University of Science and Technology, Qingdao 266590, China; qdrww92@163.com (W.R.); ytsongshr@163.com (S.S.); phywjj@163.com (J.W.); liuweier@126.com (W.L.); phywangp@163.com (P.W.)

**Keywords:** film bulk acoustic resonator, thickness shear mode, finite element method

## Abstract

A high Q factor film bulk acoustic resonator operating in thickness shear mode excited by a lateral field is described in this paper. The influence of electrode parameters on the resonator performance is studied by the finite element method. The results showed that three key electrode parameters, including the gap, length and width, played important roles in the optimization of the resonator performance. The highest Q factor of up to 643 was obtained when the parallel electrodes were designed to be 100 µm × 10 µm with the electrode gap of 10 µm. Based on the simulation results, the AlN-based film bulk acoustic resonator with a solidly mounted structure was fabricated. The testing results showed that the real device operated at the resonance frequency of 1.94 GHz with the Q factor of 405 in air, 216 in water and 102 in phosphate buffered saline solution.

## 1. Introduction

Film bulk acoustic resonators (FBARs) have been widely applied in fields related to wireless communications such as the filters in radio frequency front-end modules [1,2,3]. In addition, thanks to the high resonance frequency and high Q factor, the FBAR devices have been used as mass-loaded sensors with high sensitivity [4,5,6,7,8,9,10]. The minimum detectable mass mainly depends on the width of the resonance curve, which is inversely proportional to the Q factor [11]. Typically, the device operates in the longitudinal mode along the thickness direction. However, the longitudinal waves are considerably damped by acoustic emissions in liquids, leading to a decrease of the Q factor and a poor performance for sensors [12]. The thickness shear mode (TSM), whose particle displacement is parallel to the piezoelectric film surface, has a better resonance performance in liquids [10]. In order to obtain the shear mode resonance, many efforts have been made to grow inclined *c*-axis–oriented piezoelectric film coupled with two electrodes situated on the opposite sides of the film [13,14,15]. This method required complicated equipment and a difficult process to achieve a homogeneous *c*-axis tilt across the wafer. Moreover, the longitudinal waves may be excited by the longitudinal electric component, which makes devices operate in a mixed mode, resulting in the decrease of the Q factor in the liquid.

In this paper, a lateral field–excited (LFE) FBAR with the electrodes parallel to the piezoelectric film surface was presented. The electrode configuration was optimized to generate the lateral electric field and the TSM resonance using the finite element method (FEM). Based on the simulation results, the AlN-based FBAR with a solidly mounted structure was fabricated. The LFE FBARs require only one layer of metallization for electrodes, evidently simplifying the fabrication process compared with traditional FBARs which have to be patterned with bottom electrodes and piezoelectric films.

## 2. Device Structure and Simulation Mode

**Figure 1:**
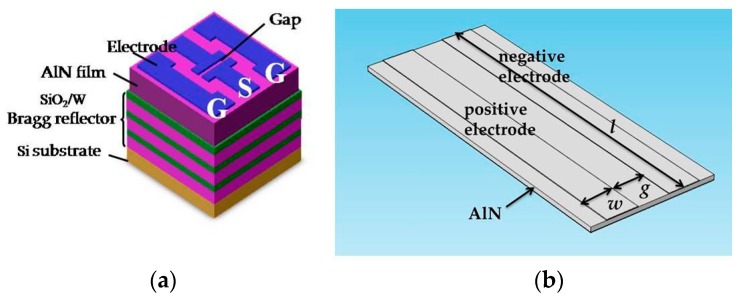
(**a**) The sketch of AlN-based solidly mounted resonator; (**b**) Geometry configuration of lateral field–excited (LFE) film bulk acoustic resonator (FBAR) for finite element method (FEM) analysis.

The basic 3D configuration of the LFE AlN-based FBAR is shown in Figure 1a. The alternating layers of SiO_2_ and W create a three-period Bragg acoustic reflector. To obtain a desired resonance frequency, the thicknesses of AlN, SiO_2_ and W were designed to be 1.5 µm, 0.50 µm and 0.37 µm, respectively. Using COMSOL Multiphysics, 3D FEM was applied to analyze a simple FBAR configuration which was composed of a *c*-axis–oriented AlN piezoelectric film and two parallel electrodes. As shown in Figure 1b, the gap, length and width of the parallel electrodes are defined as *g*, *l* and *w*. For the calculation, the two sides perpendicular to the length direction of the electrodes were applied with zero displacement constraint. The positive electrode was applied to a voltage of 1 V and the negative electrode was grounded. By conducting the harmonic analysis, the admittance curve, electric field distribution and particle displacement distribution were obtained.

## 3. Device Fabrication

All the films were deposited using the JGP800 sputtering system (ZKY Crop., Changshu, China). The detailed sputtering parameters are provided in Table 1. After the Bragg reflector was completed, the AlN film was deposited on the SiO_2_ layer by radio frequency reactive sputtering. Then the Al top electrodes were deposited on the AlN film surface and patterned by the conventional photolithography method. The pattern of the top electrode is two ports of G-S-G type in order to adapt the coplanar probes (Figure 1a). The admittance response of the device was assessed using a network analyzer (Agilent 8722, Agilent Technologies, Santa Clara, CA, USA) with a Cascade 9000TM probe station (Cascade Microtech, Inc., Beaverton, OR, USA).

## 4. The Result of FEM

### 4.1. The Influence of Electrode Gap g

**Figure 2:**
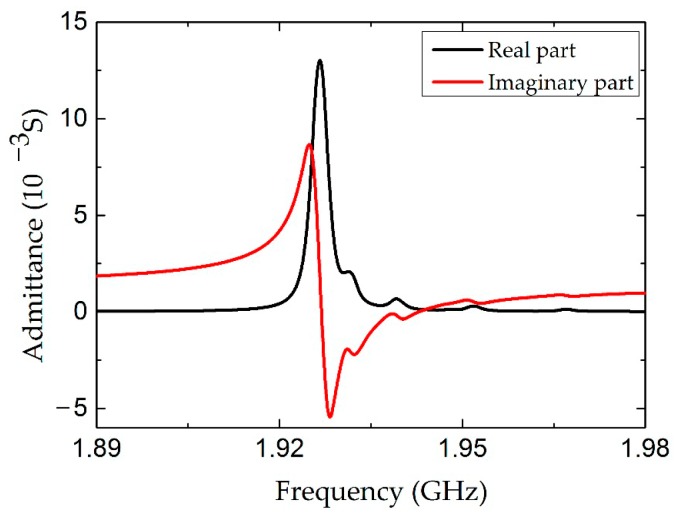
The admittance curve of the LFE FBAR device calculated by FEM.

A typical simulated admittance curve is shown in Figure 2. A clear resonance peak was found at 1.926 GHz. The Q factor of the FBAR device is evaluated as:

(1) $$Q=\frac{f_{0}}{\mathrm{BW}}$$

where the *f*_0_ is the resonance frequency, BW is the full bandwidth at half maximum of the conductance peak.

**Figure 3:**
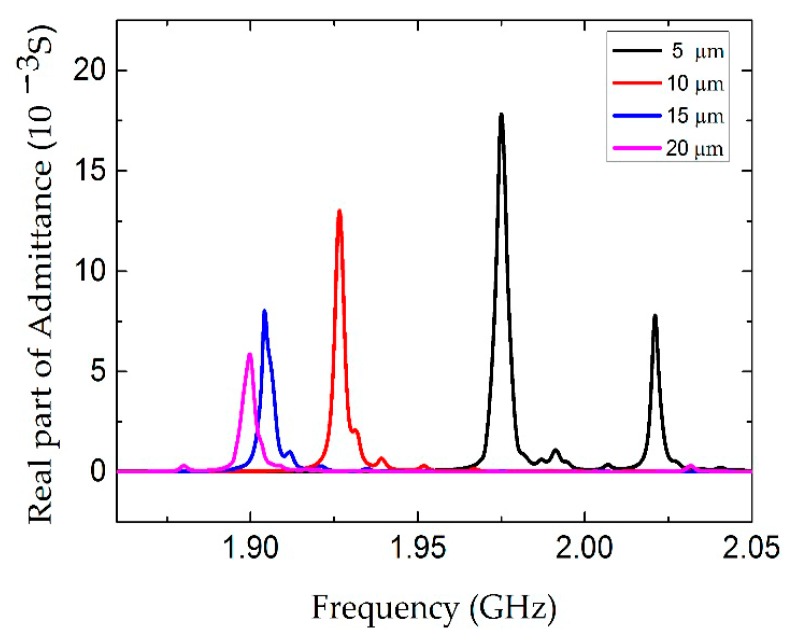
The real part of admittance curve of the LFE FBAR device for different electrode gaps *g*.

**Figure 4:**
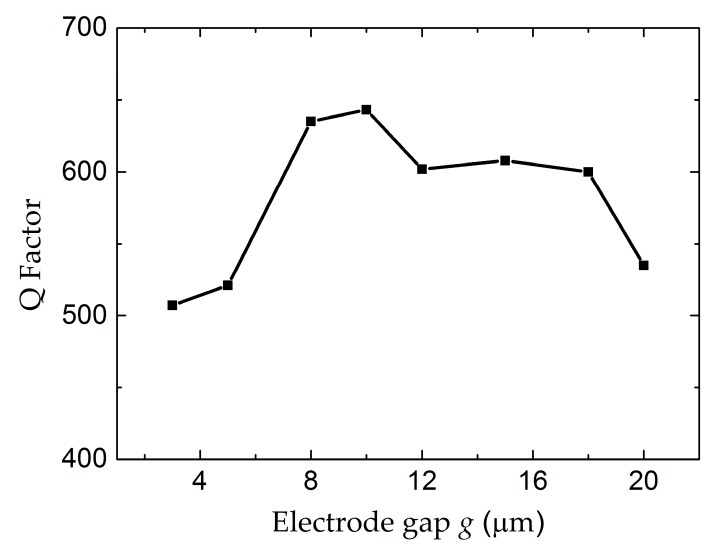
The Q factor of the LFE FBAR device as the function of the electrode gaps *g*.

**Figure 5:**
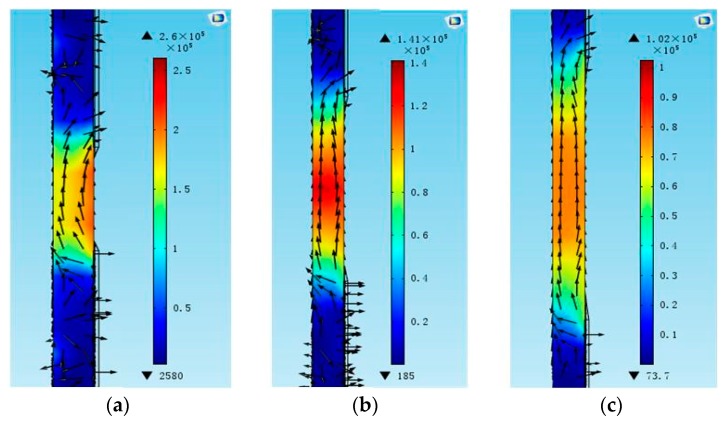
The electric field distribution of the LFE FBAR device for different electrode gaps *g* in the cross-section view. (**a**) *g* = 5 µm; (**b**) *g* = 10 µm; (**c**) *g* = 15 µm.

As the first task of the FEM simulation, the influence of the electrode gap *g* was studied. The length and width of the electrode were kept at 100 µm and 10 µm, respectively. As shown in Figure 3, the conductance curves were calculated for the devices with different electrode gaps. A spurious resonance peak appears near 2.02 GHz when the electrode gap is 5 µm, which seriously interferes with the main resonance frequency. The peak amplitude is decreased, as well as the resonance frequency, with the increase of the electrode gap. The dependence of the Q factor on the electrode gaps from 3 µm to 20 µm is shown in Figure 4. With the increase of the electrode gap, the Q factor increases and reaches the maximum when the electrode gap is 10 µm. The electric field distributions in the cross-section view were calculated as shown in Figure 5. The electric field consisting of a primarily lateral component is generated between the two electrodes, which can effectively excite the shear mode resonance in the piezoelectric film. The electric field is aligned normal to the surface near the edge of the electrode, especially for a small gap distance, leading to the excitation of a spurious wave near the main resonance frequency. However, the intensity of the lateral electric field gradually declined with the increase of the gap. Taking this fully into account, the gap between two parallel electrodes is suggested to be 10 µm.

### 4.2 The Influence of Electrode Length and Width

**Figure 6:**
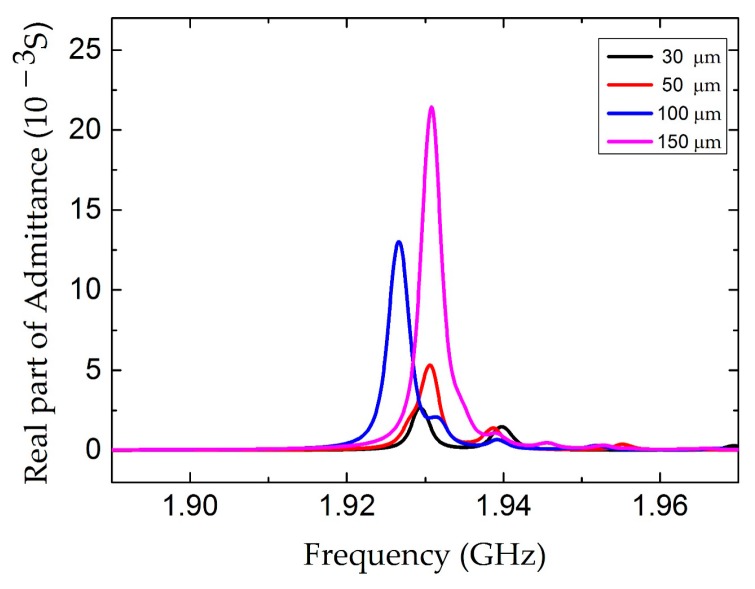
The real part of the admittance curve of the LFE FBAR device for different electrode lengths *l*.

**Figure 7:**
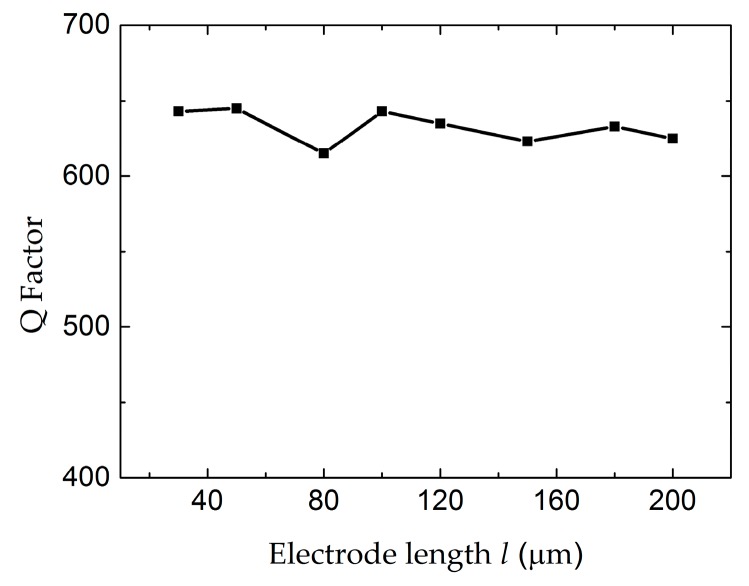
The Q factor of the LEF FBAR device as the function of the electrode lengths *l*.

**Figure 8:**
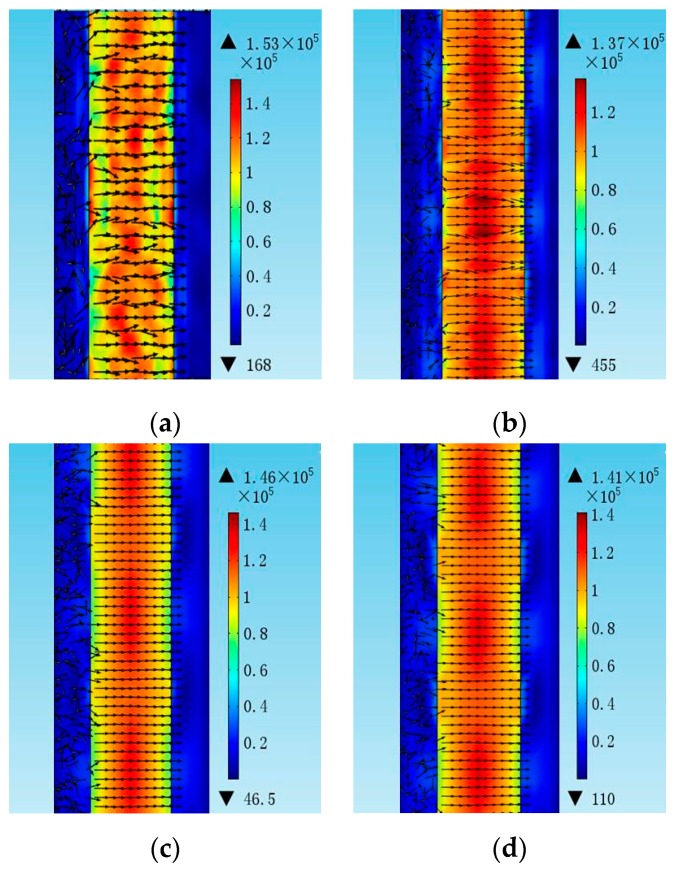
The electric field distribution of the LFE FBAR device for different electrode lengths *l* in the top view. (**a**) *l* = 30 µm; (**b**) *l* = 50 µm; (**c**) *l* = 100 µm; (**d**) *l* = 150 µm.

**Figure 9:**
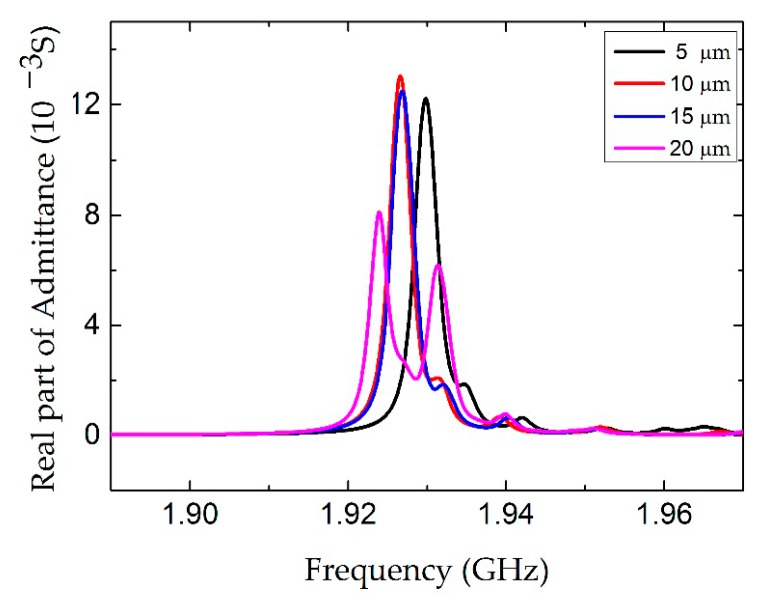
The real part of the admittance curve of the LFE FBAR device for different electrode widths *w*.

**Figure 10:**
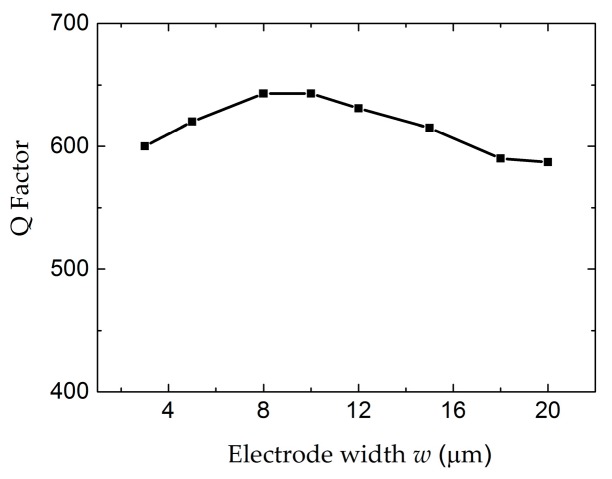
The Q factor of the LFE FBAR device as the function of the electrode widths *w*.

**Figure 11:**
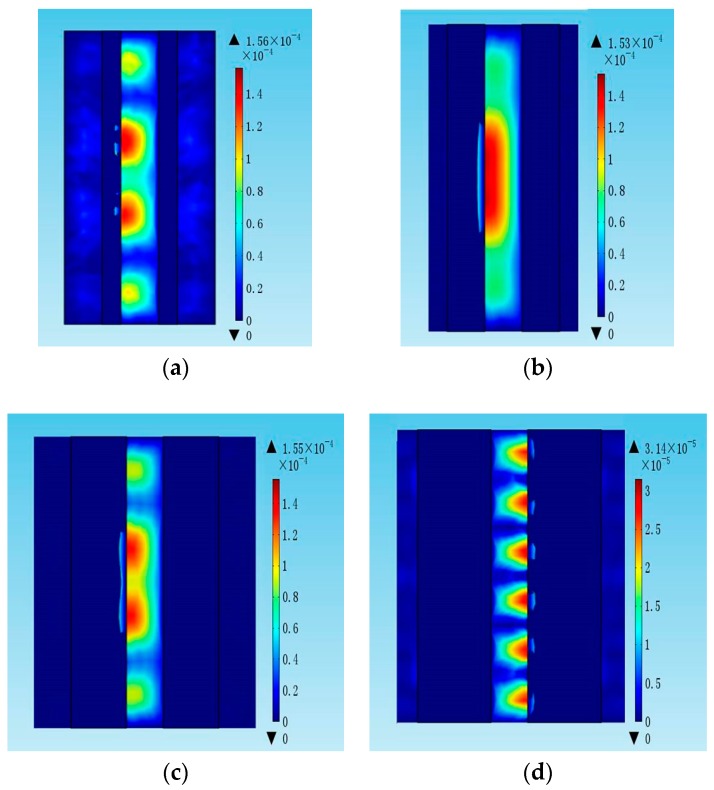
The particle displacement distribution of the LFE FBAR device for the different electrode widths w in the top view. (**a**) *w* = 5 µm; (**b**) *w* = 10 µm; (**c**) *w* = 15 µm; (**d**) *w* = 20 µm.

In order to clarify the dependence of the electrode length *l*, the conductance curves with electrode lengths from 30 µm to 150 µm were compared in Figure 6. The amplitude of the conductance curves increases with the electrode length. However, the electrode length has no obvious influence on the Q factor and the distribution of the electric field, as shown in Figure 7 and Figure 8, respectively. Figure 9 shows the conductance curves for the FBAR devices with different electrode widths *w*. The active area between the two electrodes was 100 µm × 10 µm, while the electrode widths were set from 5 µm to 20 µm. The devices show similar amplitudes of the conductance curves with the smaller electrode width. However, if the electrode width is too large relative to the electrode gap, the peak amplitude is decreased and a spurious resonance peak takes place. For all the electrode widths, the Q factors are in the range of 580–640 (Figure 10) and reach the maximum when the electrode width is 10 µm. The particle displacement distributions of the *x*-component in the top view are different, as shown in Figure 11. When the electrode width is 10 µm, the particle displacement distributes uniformly in the active area. The distribution of the lateral electric field is closely dependent on the electrodes’ configuration. These simulation results verified the theoretical analysis proposed by Zhou et al., in which the authors found the ratio of the electrode gap and the thickness of the piezoelectric layer should be no less than 2.5 or there will be a great degradation of the TSM performance [16].

## 5. The Performance of the Fabricated Device

**Figure 12:**
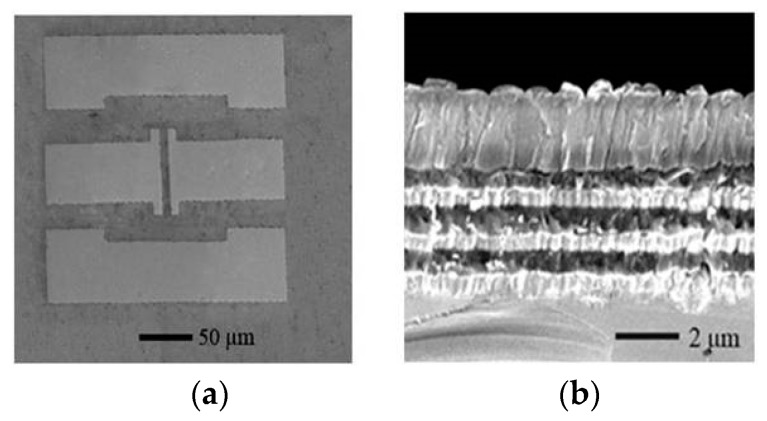
The micrograph of the LFE FBAR. (**a**) The top view; (**b**) The cross-section view.

**Figure 13:**
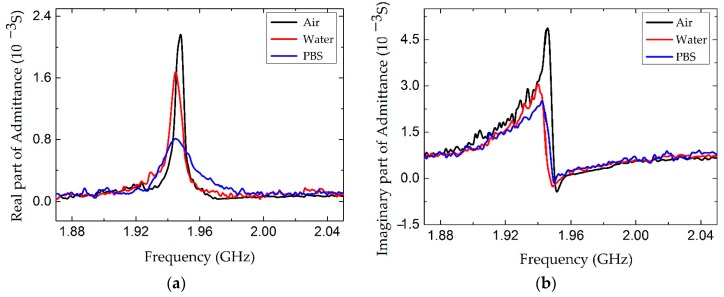
The admittance response of the LFE FBAR device. (**a**) The real part of the admittance; (**b**) The imaginary part of admittance.

A real, AlN-based FBAR device was fabricated as shown in Figure 12. Based on the FEM calculation, the structure parameters of the parallel electrodes were designed to be 10 µm × 100 µm with a gap of 10 µm. Figure 13 shows the conductance curves of the device immersed in air, water and a widely used buffer solution, phosphate buffered saline (PBS) (0.126 M NaCl, 0.1 M NaH_2_PO_4_, pH = 7.2). The resonance frequencies and the Q factors are summarized in Table 2. When the FBAR device worked in air, a resonance frequency was observed at 1.948 GHz with a Q factor of 405. The measured Q factor was smaller than the result calculated from the FEM simulation, which can be attributed to the energy absorption and interface scattering in the sputtered films. The damping effect of the liquids resulted in a frequency shift of about 4 MHz and 23.5% in water, 63.3% in PBS solution attenuation in the conductance amplitude. Consequently, the Q factors decreased to 216 and 102 in the water and PBS solution, respectively. It is concluded that the resonance performances of the LFE FBAR were greatly influenced by the conductivity of the contacting solutions, which is a limitation of the devices for bio-sensing applications. However, although the Q factor of the LFE FBAR dropped to 102 in the buffer solution, the devices have promising applications in biochemical detection. In comparison, Zhang et al. reported a longitudinal-mode FBAR working with a Q factor of only 40 in water [17]. Moreover, the device coated with TiO_2_ has been successfully used to detect the 10 mM K^+^ ions in the K_2_CO_3_ solution [11].

## 6. Conclusions

The electric and resonant characteristics of the LFE FBAR were analyzed using FEM to optimize the parallel electrode configuration. The results predicted that the best performance was obtained when the parallel electrode configuration was designed to be 100 µm × 10 µm with a gap of 10 µm. The admittance response of the real device showed an obvious shear mode resonance at 1.94 GHz and a Q factor of 405 in air, 216 in water and 102 in PBS solution, respectively. The devices have promising applications for biochemical sensors working in liquid.

## Figures and Tables

**Table 1:** The detailed sputtering parameters for each layer.

Layer	Power (W)	Pressure (Pa)	Gas Flow (sccm)	Substrate Temperature (°C)
AlN	RF 150	0.6	Ar:8 N_2_:6	300
SiO_2_	RF 200	0.3	Ar:5	150
W	DC 100	0.3	Ar:5	150

**Table 2:** The resonance frequency and Q factor in air, in water and in phosphate buffered saline (PBS) solution.

Parameters	Air	Water	PBS
*f*/GHz	1.948	1.944	1.944
Q	405	216	102
